# *Schistosoma mansoni* coactivator associated arginine methyltransferase 1 (SmCARM1) effect on parasite reproduction

**DOI:** 10.3389/fmicb.2023.1079855

**Published:** 2023-02-24

**Authors:** Fernanda Sales Coelho, Sandra Grossi Gava, Luiza Freire Andrade, Juliana Assis Geraldo, Naiara Clemente Tavares, Felipe Miguel Nery Lunkes, Renata Heisler Neves, José Roberto Machado-Silva, Raymond J. Pierce, Guilherme Oliveira, Marina Moraes Mourão

**Affiliations:** ^1^Grupo de Pesquisa em Helmintologia e Malacologia Médica, Instituto René Rachou, Fundação Oswaldo Cruz—FIOCRUZ, Belo Horizonte, Minas Gerais, Brazil; ^2^Department of Clinical Research, London School of Hygiene and Tropical Medicine, London, United Kingdom; ^3^Laboratório de Helmintologia Romero Lascasas Porto, Departamento de Microbiologia, Imunologia e Parasitologia, Faculdade de Ciências Médicas, Universidade do Estado do Rio de Janeiro, Rio de Janeiro, Brazil; ^4^Université de Lille, CNRS, Inserm, CHU Lille, Institut Pasteur de Lille, U1019—UMR 9017—CIIL—Centre d’Infection et d’Immunité de Lille, Lille, France; ^5^Instituto Tecnológico Vale, Belém, Pará, Brazil

**Keywords:** *Schistosoma mansoni*, coactivator associated arginine methyltransferase 1, SmCARM1, epigenetics, reproduction

## Abstract

**Introduction:**

The human blood fluke parasite *Schistosoma mansoni* relies on diverse mechanisms to adapt to its diverse environments and hosts. Epigenetic mechanisms play a central role in gene expression regulation, culminating in such adaptations. Protein arginine methyltransferases (PRMTs) promote posttranslational modifications, modulating the function of histones and non-histone targets. The coactivator-associated arginine methyltransferase 1 (CARM1/PRMT4) is one of the *S. mansoni* proteins with the PRMT core domain.

**Methods:**

We carried out *in silico* analyses to verify the expression of SmPRMTs in public datasets from different infection stages, single-sex versus mixed-worms, and cell types. The SmCARM1 function was evaluated by RNA interference. Gene expression levels were assessed, and phenotypic alterations were analyzed *in vitro*, *in vivo*, and *ex vivo*.

**Results:**

The scRNAseq data showed that SmPRMTs expression is not enriched in any cell cluster in adult worms or schistosomula, except for Sm*carm*1 expression which is enriched in clusters of ambiguous cells and Sm*prmt*1 in NDF+ neurons and stem/germinal cells from schistosomula. Sm*prmt*1 is also enriched in S1 and late female germ cells from adult worms. After dsRNA exposure *in vitro*, we observed a Sm*carm*1 knockdown in schistosomula and adult worms, 83 and 69%, respectively. Sm*carm*1-knockdown resulted in reduced oviposition and no significant changes in the schistosomula or adult worm phenotypes. *In vivo* analysis after murine infection with Sm*carm*1 knocked-down schistosomula, showed no significant change in the number of worms recovered from mice, however, a significant reduction in the number of eggs recovered was detected. The *ex vivo* worms presented a significant decrease in the ovary area with a lower degree of cell differentiation, vitelline glands cell disorganization, and a decrease in the testicular lobe area. The worm tegument presented a lower number of tubercles, and the ventral sucker of the parasites presented a damaged tegument and points of detachment from the parasite body.

**Discussion:**

This work brings the first functional characterization of SmCARM1 shedding light on its roles in *S. mansoni* biology and its potential as a drug target. Additional studies are necessary to investigate whether the reported effects of Sm*carm*1 knockdown are a consequence of the SmCARM1-mediated methylation of histone tails involved in DNA packaging or other non-histone proteins.

## Introduction

Schistosomiasis is a neglected parasitic disease with high rates of morbidity and mortality, producing significant economic losses, especially occurring in developing countries (Adepoju, 2020; WHO, 2022). The disease is caused by parasites of the genus *Schistosoma*, which have well-known and complex life cycles. Parasites require numerous and diverse mechanisms to change their anatomy and physiology and enable adaptation to different environments and hosts (Colley et al., 2014). Such adaptations are orchestrated by intense regulation of gene expression in which epigenetics mechanisms play a central role.

Schistosomiasis treatment relies on a single drug, praziquantel. The drug is effective with low side effects, but resistance has been described (Botros and Bennett, 2007; Doenhoff et al., 2008; Coeli et al., 2013). Since epigenetic proteins are promising targets for parasitic drug development, especially in helminths (Marek et al., 2013; Melesina et al., 2015) and a wide variety of epi-drugs are available; the piggy-back strategy might expedite the discovery of new specific leads and might allow the development of a new drug.

Eukaryotic chromatin remodeling promotes important parasite transcriptional changes and is mediated by nucleosome arrangements, DNA methylation, histone variants, non-coding RNAs, and posttranslational modifications (PTMs) of histones (Lee et al., 2010; Margueron and Reinberg, 2010). In this context, histone-modifying enzymes (HMEs) mediate the addition or removal of methyl, acetyl, or phosphate groups, among others, in the histone tails, remodeling the chromatin structure and altering the access to specific DNA segments (Jenuwein and Allis, 2001; Margueron and Reinberg, 2010). Histone methylation is an important PTM process that plays a crucial role in transcriptional control, splicing, DNA repair, and signaling (Dillon, 2004).

Protein arginine methyltransferases (PRMTs) promote the transference of methyl groups to the arginine residues of histones and other proteins (Lee et al., 1977). The eukaryotic PRMTs are classified into three different types. Type-I enzymes (PRMT1, PRMT3, PRMT4, PRMT6, and PRMT8) catalyze the formation of monomethyl arginine (MMA) and asymmetric dimethylarginine (aDMA), type-II enzymes (PRMT5 and PRMT9) form MMA and symmetric dimethylarginine (sDMA), while type-III enzymes (PRMT7) catalyze only MMA formation (Hartley and Lu, 2020). A type-IV enzyme catalyzes the monomethylation of the internal guanidino nitrogen atom and is reported only for yeast (RMT2; Bedford and Clarke, 2009). The posttranslational arginine methylation promoted by PRMTs modulates the function of histone proteins and non-histone targets, like transcription factors, coactivators, corepressors, and RNA-binding proteins. Accordingly, PRMTs regulate diverse cellular processes, including cell cycle, transcription, splicing, translation, signal transduction, and DNA damage and repair (Hwang et al., 2021).

Histone methylation processes play a role in *S. mansoni* development, as different life stages of the parasite present specific methylation profiles and the pharmacological inhibition of methyltransferases impacts the transition of miracidium to sporocyst (Roquis et al., 2018). *Schistosoma mansoni* possesses five proteins with the PRMT core domain (Smp_029240, Smp_025550, Smp_070340, Smp_171150, and Smp_337860; Padalino et al., 2018). The protein arginine methyltransferase 1 (SmPRMT1, Smp_029240) was the first and only characterized PRMT in *S. mansoni*. Mansure et al. demonstrated that SmPRMT1 methylates histone H4, besides SMYB1 and SmSmD3, proteins likely to be involved in RNA metabolism; suggesting the involvement of SmPRMT1 in chromatin remodeling and RNA editing and transport (Mansure et al., 2005). Additionally, Diao and collaborators demonstrated the immunogenic role of PRMT1 from *Schistosoma japonicum* pointing out this enzyme as a promising molecule for vaccine development (Diao et al., 2014).

The coactivator-associated arginine methyltransferase 1 (CARM1), also known as protein arginine methyltransferase 4 (PRMT4), regulates the expression of genes related to cell cycle progression and autophagy (El Messaoudi et al., 2006; Shin et al., 2016). CARM1 methylates the arginines 17 and 26 of histone H3 (H3R17, H3R26) and also functions as a coactivator in non-nuclear receptor systems like NF-κB, p53, INF-γ, MEF2C, and β-Catenin reviewed in Yue et al. (2007). In mice, CARM1-knockout embryos present small size and perinatal death (Yadav et al., 2003). Due to the important roles attributed to CARM1 orthologs and the lack of functional information in the *S. mansoni* parasite, here we aim at contributing to the functional characterization of this enzyme with a focus on schistosomula and adult stages, to shed light on the roles of SmCARM1 (Smp_070340) in *S. mansoni* biology and its potential as a drug target.

## Materials and methods

### Ethics statement

All procedures for the scientific use of animals were reviewed according to the Brazilian ethical guidelines (Law 11,794/08) and approved by the Ethics Committee for Animal Use (CEUA) of the Oswaldo Cruz Foundation under license numbers LW13/13 and LW12/16.

### Reagents

The following reagents were used for parasite culture, Glasgow Minimum Essential Medium (GMEM), triiodothyronine, lactalbumin, HEPES, MEM vitamin solution, Schneider’s Insect Medium, hypoxanthine, and hydrocortisone were purchased from Sigma-Aldrich. Penicillin/streptomycin, RPMI 1640 medium, and Fetal Bovine Serum (FBS) were from Gibco; glucose from VETEC; TRIzol Reagent from Invitrogen.

All primers were purchased from IDT, Sm*carm*1-dsRNA_F: taatacgactcactatagggCATGGCATGGATCTAACTGC, Sm*carm*1-dsRNA_R: taatacgactcactatagggTGTTGTTGTTGCTGTTGTGC, Sm*carm*1-qPCR_F: TGCTGTTGAAGCATCTAATATGG, Sm*carm*1-qPCR_R: ATAATGACATCCACTGGTTCG; Sm*cox*I (Smp_900000) Sm*cox*I-qPCR_F: TACGGTTGGTGGTGTCACAG, Sm*cox*I-qPCR_R: ACGGCCATCACCATACTAGC; GFP-dsRNA_F: taatacgactcactatagggTCTTCAAGTCCGCCATG, GFP-dsRNA_R: taatacgactcactatagggTGCTCAGGTAGTGGTTGTC. The sequences in lowercase correspond to the T7 promoter sequence added to the 5′-end in primers designed for dsRNA synthesis.

### Parasites

The “Lobato Paraense” snail facility at the René Rachou Institute—FIOCRUZ provided cercariae of *S. mansoni* (LE strain). The parasite cycle is maintained throughout passages between hamsters (*Mesocricetus auratus*) and snails (*Biomphalaria glabrata*).

As previously described, cercariae were mechanically transformed into schistosomula (Milligan and Jolly, 2011). Schistosomula were cultured in GMEM supplemented with 0.2 μM triiodothyronine; 0.1% glucose; 0.1% lactalbumin; 20 mM HEPES; 0.5% MEM vitamin solution; 5% Schneider’s Insect Medium; and 0.5 μM hypoxanthine, 1 μM hydrocortisone, 1% penicillin/streptomycin, and 2% heat-inactivated FBS.

Hamsters (*M. auratus*) were infected with cercariae and subjected to perfusion (Pellegrino and Siqueira, 1956) after 45 days for obtaining adult worms. Males and females were washed, separated manually, and cultured in Roswell Park Memorial Institute 1640 (RPMI 1640) medium supplemented with 2% penicillin/streptomycin and 10% heat-inactivated FBS.

### *In silico* analyses

To verify the expression data of SmPRMTs in the different cell types of adult *S. mansoni* worms, single-cell RNAseq (scRNAseq) data were obtained from the Gene Expression Omnibus database (GEO, https://www.ncbi.nlm.nih.gov/geo/, BioProject PRJNA611783, SRA SRP252217; https://www.collinslab.org/schistocyte/; Wendt et al., 2020). We also verified Sm*carm*1 expression in clusters of cells identified in schistosomula at the cellxgene platform^1^ (Diaz Soria et al., 2020). The RDS file containing the expression data in the different cell types was loaded in the R software (v4.1.2; R Core Team, 2020) using the Seurat package (v4.1.1; Satija et al., 2015) and used to build a heatmap with the package ComplexHeatmap (v2.10.0; Gu et al., 2016). Additionally, we checked the SmPRMTs expression in a publicly available RNAseq dataset (Protasio et al., 2017) retrieved from WormBase ParaSite as counts of aligned reads per run per gene. Differential expression analysis was carried out using DESeq2 (v. 2_1.38.2; *padj* < 0.05; Love et al., 2014) and we used the pheatmap package (v1.0.12; Kolde, 2015) in R (v4.1.2; R Core Team, 2020) to construct a heatmap representing the log2 fold change for 18-, 28-, 35-, and 38-day post-infection (dpi) relative to 21 dpi.

### Double-stranded RNA synthesis and parasite exposure

The Sm*carm*1 coding sequence was obtained from the GeneDB database.^2^ The T7 promoter sequence was added to the 5′-end of primers designed to amplify a template of 569 bp for double-stranded RNAs (dsRNAs) synthesis. A fragment of 360 bp from the green fluorescent protein (GFP) cloned in the pCRII plasmid vector, was used as non-schistosome RNA interference (RNAi) control. The PCR amplified fragments were purified with the QIAquick Gel Extraction KIT (QIAGEN) and used for dsRNA synthesis using the T7 RiboMAX Express RNAi System kit (Promega) according to the supplier’s protocol; except for the time of reactions which was changed to 16 h. DsRNAs annealing and integrity were verified in 1% agarose gel electrophoresis, and the quantification was estimated in a Nanodrop Spectrometer ND-1000 (Thermo Fischer Scientific).

Approximately 2,000 schistosomula were exposed to 100 nM of dsRNAs (Sm*carm*1 or GFP), immediately after mechanical transformation, in 24-well plate and incubated for 7 days at 37°C, 5% CO_2_, and 95% humidity with 2 ml of GMEM supplemented as previously mentioned.

Eight adult worms (males and females, separately) were placed in 100 μl of RPMI 1640 medium with 25 μg of dsRNA. The worms were electroporated with specific Sm*carm*1-dsRNA or unspecific GFP-dsRNA into 4 mm cuvettes at 125 V for 20 ms and cultivated in 24-well plate with 1 ml RPMI 1640 medium supplemented with 10% heat-inactivated FBS and 2% penicillin/streptomycin. Similarly, to count the number of eggs laid, eight worm pairs were electroporated and cultured in six-well plate and the medium was changed daily.

### RNA extraction, cDNA synthesis, and qPCR analysis

Seven days after dsRNA exposure, 1,000 schistosomula were separated for RNA extraction and relative expression analysis by quantitative real-time PCR (RT-qPCR). *Schistosoma mansoni* cytochrome C oxidase I gene (Sm*cox*I—Smp_900000) was used as the internal control gene. RNA extractions were performed using the TRIzol Reagent method followed by purification with the RNeasy Mini Kit (Qiagen), according to the manufacturer’s guidelines. RNA samples were treated with the TURBO DNA-free kit (Ambion) to remove residual genomic DNA, quantified using the Nanodrop Spectrometer ND-1000, and stored at −70°C.

For adults, for 7 days, two worm pairs per day were removed and macerated with TRIzol Reagent for RNA extraction as described previously. Experiments were performed in four biological replicates.

The cDNAs were synthesized with equal amounts of the extracted RNAs using the SuperScript II Reverse Transcriptase (Invitrogen), with oligo(dT)18 following the manufacturer’s protocol. Primers for qPCR analysis were designed using the Primer 3 program.^3^ Primer efficiencies were estimated by titration analysis to be 100 ± 5% (data not shown), and the specificity was verified by the melting curve. qPCR reactions were performed on 7500 Real-Time PCR System (Applied Biosystems) with SYBR Green PCR Master Mix (Applied Biosystems) and 200 nM of each primer in a final volume of 25 μl. Internal controls to evaluate genomic DNA contaminations (RNA samples) and reagent purity (no cDNA) were included in all analyses. The 2^−ΔΔCt^ method (Livak and Schmittgen, 2001) was used for relative quantification and normalized with Sm*cox*I. Transcript levels were expressed as a percentage of difference relative to the unspecific (GFP) or negative control.

### Phenotypic evaluation of schistosomula and adult worms

Schistosomula cultures were daily observed by inverted light microscopy (ABO 100, ZEISS) to verify viability and phenotypic changes, such as movement, color, and tegument integrity.

To evaluate the motility of the worms, we capture worm movement (eight males or females separately) for 1 min and 30 s for 7 days using the WormAssay software (Marcellino et al., 2012), in six replicates. Additionally, to verify the influence of SmCARM1 on oviposition, eggs laid in the media were counted daily in the cultures containing worm pairs, in five replicates.

### *In vivo* experiments

After schistosomula exposure to dsRNAs for 2 days, 300 schistosomula were subcutaneously inoculated in Swiss mice (*Mus musculus*). Each experimental group consisted of at least six mice in three independent biological replicates. Before the infection, the Sm*carm*1 transcript levels in schistosomula were checked by qPCR, as described above. Schistosomula exposed to unspecific dsRNA-GFP and untreated parasites were used as controls. After 37 days adult worms were recovered by perfusion (Pellegrino and Siqueira, 1956). After perfusion, the worms recovered from the mice were separated into males and females and counted. Mouse livers were removed and individually weighed, crushed with a scalpel, and treated overnight with 10% KOH, for subsequent egg counting (Tavares and Mourão, 2021).

### Morphometric and confocal analysis

The adult worms recovered after 37 days of mouse infection were fixed and stored in Alcohol-Formalin-Acetic Acid (AFA, 95% ethanol, 3% formaldehyde, and 2% glacial acetic acid), at room temperature, and stained with 2.5% chloride carmine, dehydrated in alcoholic series (70, 90%, and absolute), clarified in methyl salicylate with Canadian balsam (1:2), and prepared as whole-mounts (Neves et al., 1998). We analyzed at least six males and six females recovered from mice infected with schistosomula previously exposed to Sm*carm1*-, GFP-dsRNA, or untreated, from the three biological replicates.

Computer images (Image Pro Plus, Media Cybernetics), from male and female worms captured by a camera (640/480 pixels, RGB) coupled to a light microscope (BX50, Olympus), were used for morphometric analyses. We evaluated the number and area of testicular lobes, ovary area, presence of tubercles, presence of eggs and vitelline glands, and integrity of the tegument (Neves et al., 2004).

Whole mounts of male and female worms were also analyzed under confocal laser scanning microscopy (LSM-410, Zeiss) using a 543 nm laser and a BP560-615 IR filter, in reflected mode. We examined male (testicular lobes, seminal vesicle) and female (yolk glands, ovary, uterus, and ootype) reproductive systems, as well as the integrity of the tegument and the shape of the oral and ventral suckers.

### Statistical analysis

All statistical analyses were performed using GraphPad Prism, v. 7 for Windows (GraphPad Software, www.graphpad.com). After verifying for outliers, using the ROUT method (*Q* = 1%; Motulsky and Brown, 2006), and applying normality test analysis to check if the data follows a Gaussian distribution, statistical analyses used the Mann–Whitney test (Wilcoxon-Sum of Ranks, *p* < 0.05), Paired or Unpaired *t*-test (*p* < 0.05), as described in the respective figure legends.

## Results

### PRMTs expression in *Schistosoma mansoni*

At first, we aimed at analyzing existing RNAseq and single-cell RNAseq data available in the WormBase ParaSite, the Gene Expression Omnibus database, and cellxgene platform. All results for differential expression analysis retrieved for PRMTs (Smp_025550—SmPRMT7, Smp_029240—SmPRMT1, Smp_070340—SmCARM1, Smp_171150—SmPRMT6, and Smp_337860—SmPRMT3) are available in Supplementary Table S1. In this analysis, we found that PRMTs identified in the *S. mansoni* genome present a significative decrease in the expression profiles for male adult worms from single-sex infections, from 18- to 21-dpi (Supplementary Figure S1), except for Sm*prmt*6. Females from unisexual or mixed infections presented a similar expression profile of Sm*carm*1 from 21- to 35-dpi, with a significant reduction in expression. However, only females in the presence of male worms return to the transcript levels of those observed for 21 dpi, which is observed for all PRMTs, specially Sm*prmt*6. Whereas, in males, single or mixed infections do not differently impact the Sm*carm*1 expression profile.

**Figure 1:**
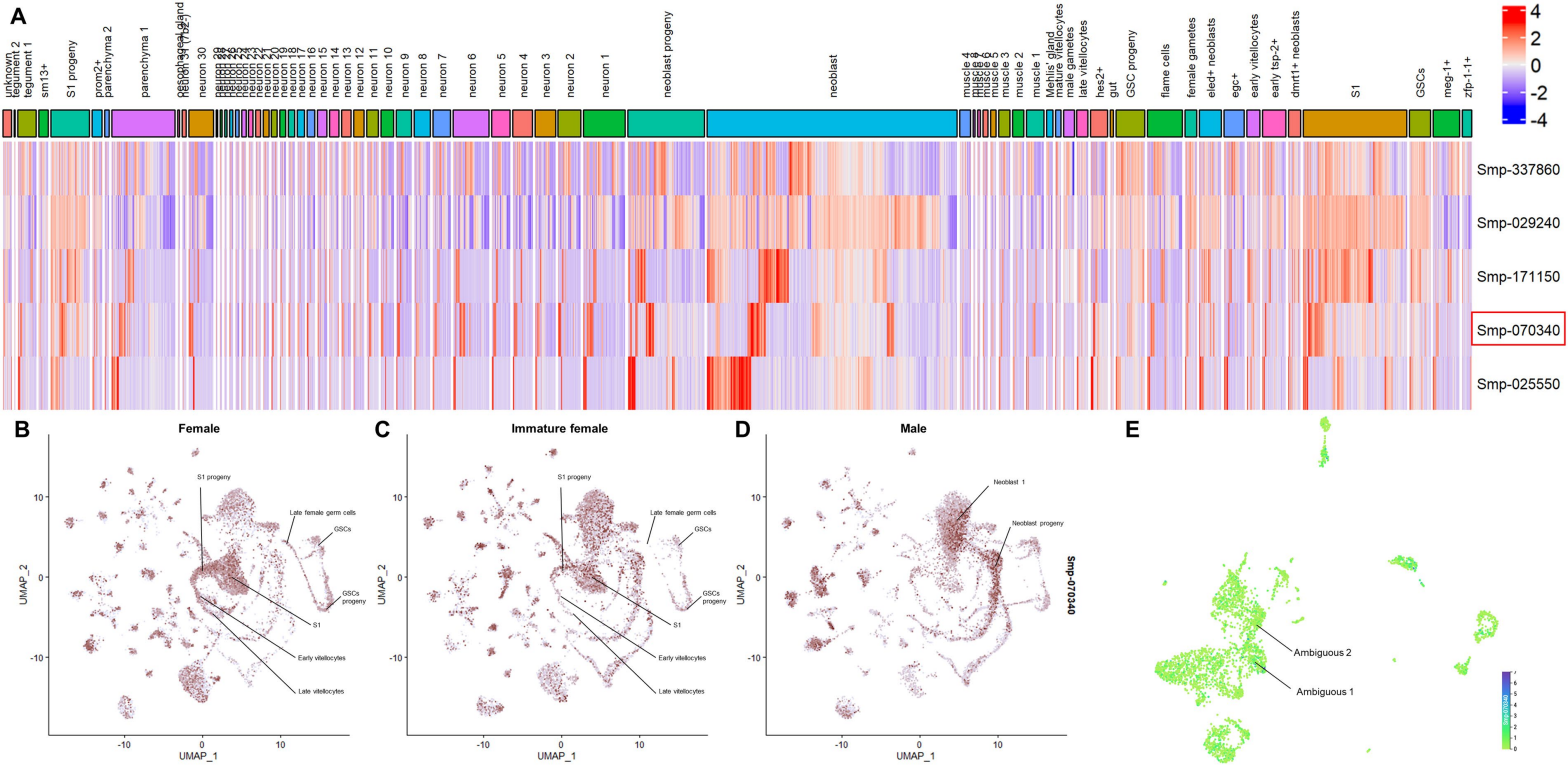
SmPRMTs expression in different cell types identified in *Schistosoma mansoni* adult worms. **(A)** SmPRMTs expression in different cell types identified in *S. mansoni* adult worms. The columns indicate the different cell types identified in adult worms and the lines represent the different SmPRMTs analyzed. The color scale indicates higher (red) or lower (blue) expression of transcripts. Sm*carm*1 (Smp_070340) is highlighted by a red box. **(B–D)** UMAP projections depicting the expression profiles of Sm*carm*1 in different cell clusters in **(B)** female, **(C)** immature female, and **(D)** male *S. mansoni* adult worms. Expression values are normalized and represented by colors (gray = not expressed, red = expressed). **(E)** UMAP projections depicting the expression profiles of Sm*carm*1 in 13 transcriptionally distinct cell types in schistosomula. Expression values are normalized and represented by colors ranging from light green (not expressed) to purple (highly expressed).

The analysis of the scRNAseq data provided by Wendt et al. (2020) and Diaz Soria et al. (2020) allowed the assessment of the expression of *S. mansoni* SmPRMTs in different cell types of *S. mansoni* adult worms and schistosomula, respectively, (Figure 1). Sm*prmt*1 (Smp_029240) expression is enriched in Supplementary Figure S1 and late female germ cells, whereas expression of the other SmPRMTs, including Sm*carm*1, is not enriched in any cell cluster. In schistosomula, Sm*prmt*1 expression is enriched in NDF+ neurons and stem/germinal cells, and Sm*carm*1 expression is enriched in clusters of ambiguous cells (Figure 1E), while expression of the other SmPRMTs is not enriched in any cell cluster.

### *In vitro* Sm*carm*1 knockdown on schistosomula

**Figure 2:**
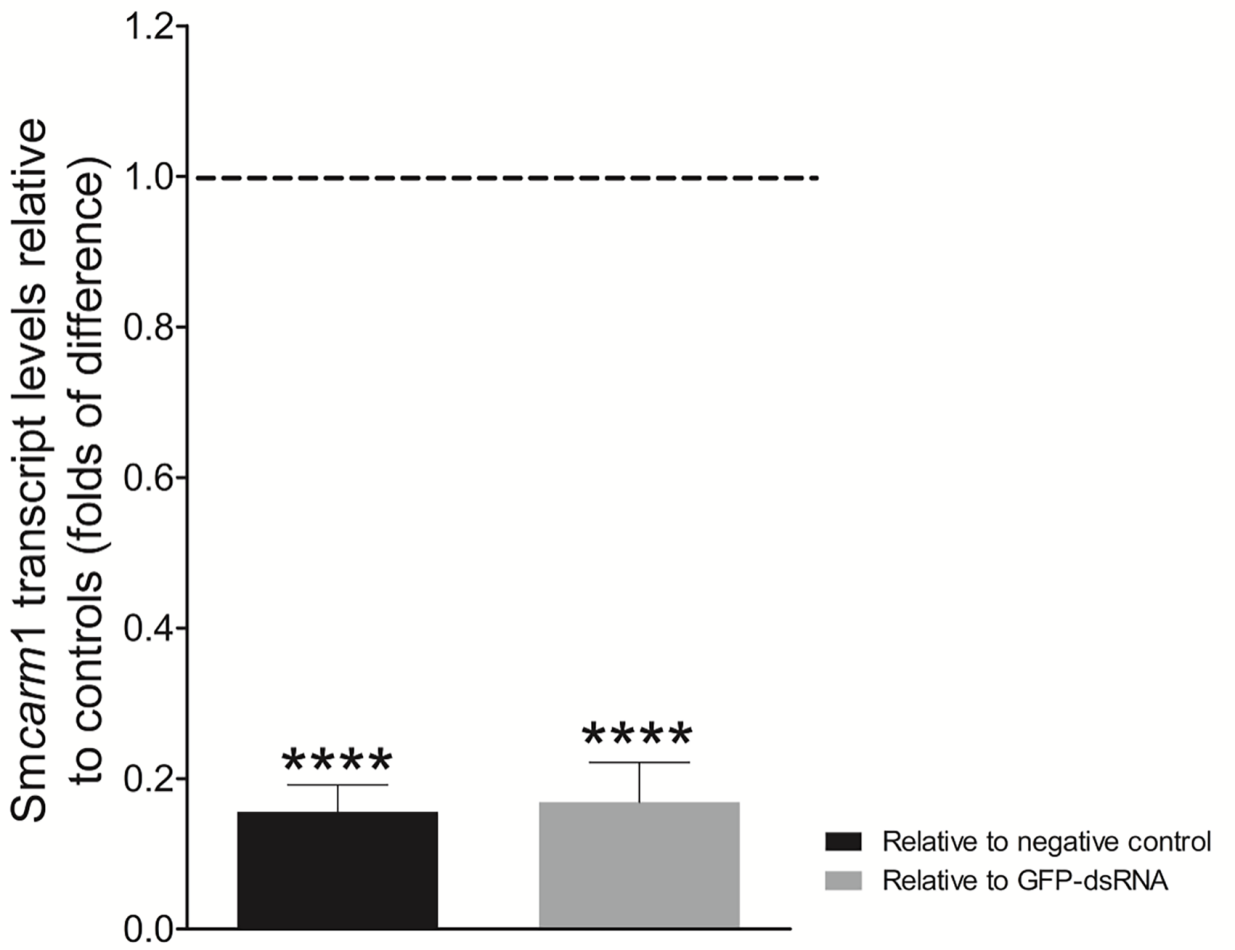
Sm*carm*1 transcript levels in schistosomula after dsRNA exposure. Bar graph depicting the Sm*carm*1 transcript levels in schistosomula after 7 days of exposure to Sm*carm*1-dsRNA relative to negative (black) or unspecific GFP-dsRNA (gray). Data are represented as mean fold-difference (±SE) relative to controls (dashed line). Mann–Whitney test. ^****^p < 0.0001.

There is a lack of functional characterization of PRMTs in the *S. mansoni* parasite. Since important roles have been attributed to CARM1 in other organisms, we further investigated whether SmCARM1 had effects on worm biology, so to that end we knocked-down the Sm*carm*1 transcripts by RNAi experiments. After 7 days of dsRNA exposure, schistosomula presented an average 83% reduction in Sm*carm*1 transcript levels (Figure 2) compared to the controls. No significant changes were observed in viability or evaluated phenotypes, such as movement, color, tegument integrity, or area in *in vitro* cultivated parasites (data not shown).

### *In vitro* Sm*carm*1 knockdown on adult worms

**Figure 3:**
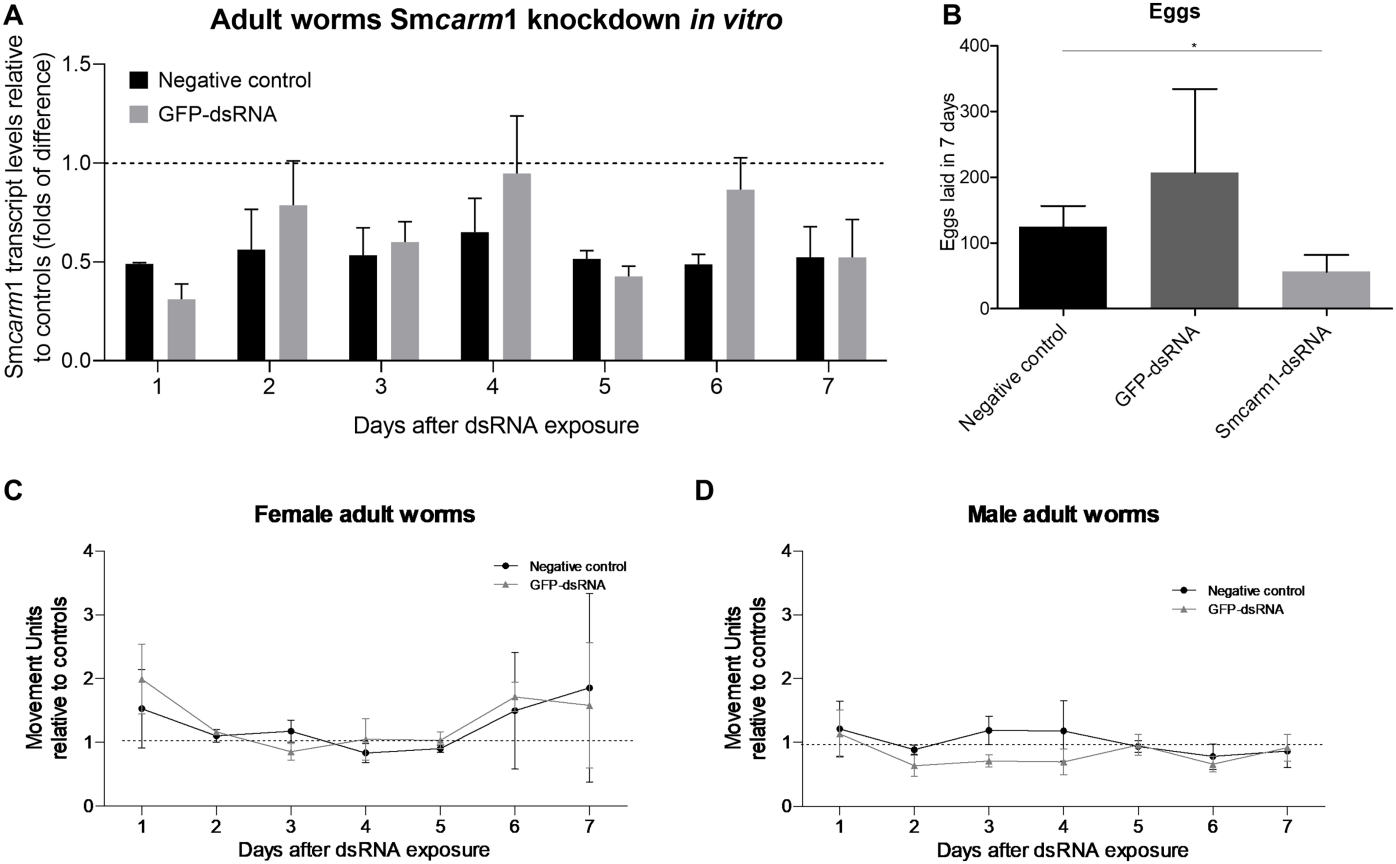
Sm*carm*1 knockdown in adult worms *in vitro*. **(A)** Sm*carm*1 transcript levels in adult worms after dsRNA exposure. Bar graph depicting the Sm*carm*1 transcript levels in adult worms during 7 days of exposure to Sm*carm*1-dsRNA relative to negative (black) or unspecific GFP-dsRNA (gray). Data are represented as mean fold-difference (±SE) relative to controls (dashed line). Mann–Whitney test, *N* = 4. **(B)** Bar graph depicting the number of eggs laid during 7 days for adult worms from the negative control (black) and after electroporation with GFP-dsRNA (dark gray) or Sm*carm*1-dsRNA (light gray). Paired *t*-test (^*^p < 0.05), *N* = 5. Movement units from **(C)** female and **(D)** male adult worms knocked-down for Sm*carm*1 normalized with negative (black) or unspecific control (gray). The dotted line represents normalized movement units in the control. Two-way ANOVA, *N* = 6.

Adult worms electroporated with Sm*carm*1-dsRNA showed up to a 69% reduction in Sm*carm*1 transcript levels on the first day after electroporation (Figure 3A). Sm*carm*1-dsRNA knockdown resulted in a significant reduction of 54.9% in egg laying compared to the negative control (Figure 3B), although no relevant changes were observed in the movement of females or males for 7 days (Figures 3C,D).

### Effect of Sm*carm*1 knockdown on worm development in a murine model

**Figure 4:**
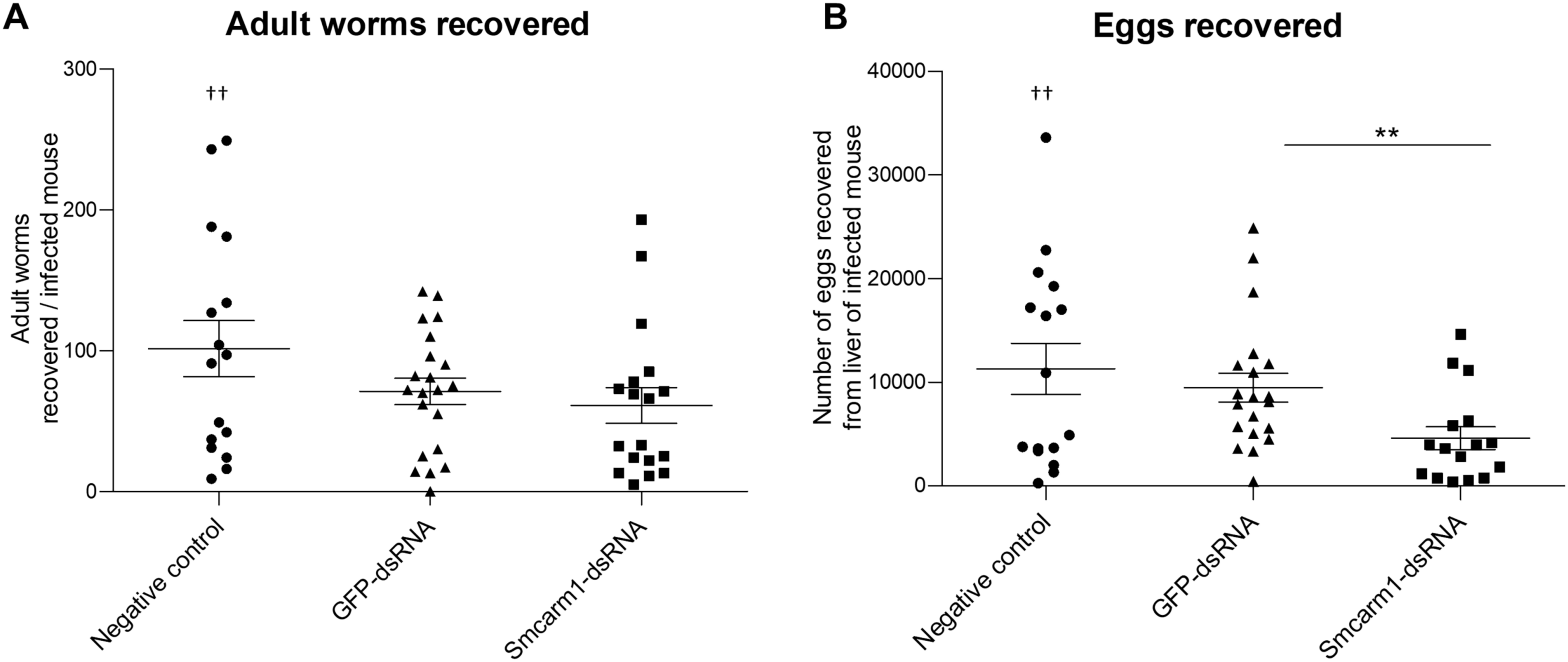
Analysis of *ex vivo* adult worms and evaluation of egg recovery from mice liver. Adult worms recovered from mice infected with schistosomula from negative control (●), unspecific GFP-dsRNA (▲), and Sm*carm*1-dsRNA (■). Each symbol in the chart represents **(A)** worm counts per mouse or **(B)** egg counts per mouse liver. Dead mouse is represented (†) above the plotted data. The horizontal lines represent the median values per group. Data were generated from three independent experiments and statistically analyzed using the Mann–Whitney test. ^**^p < 0.01.

The conclusions drawn from *in vitro* experiments could be limited since parasites are not challenged by a real biological system. Therefore, we sought to investigate the role of SmCARM1 on *in vivo* infections. Before infection, the reduction of Sm*carm*1 transcript levels in schistosomula was confirmed by RT-qPCR (Supplementary Figure S2). After perfusion, there was no significant change in the number of worms, male or female, recovered from mice infected with Sm*carm*1 knocked-down schistosomula (Figure 4A). However, there was a significant reduction in the number of eggs recovered from the livers of these mice (Figure 4B).

**Figure 5:**
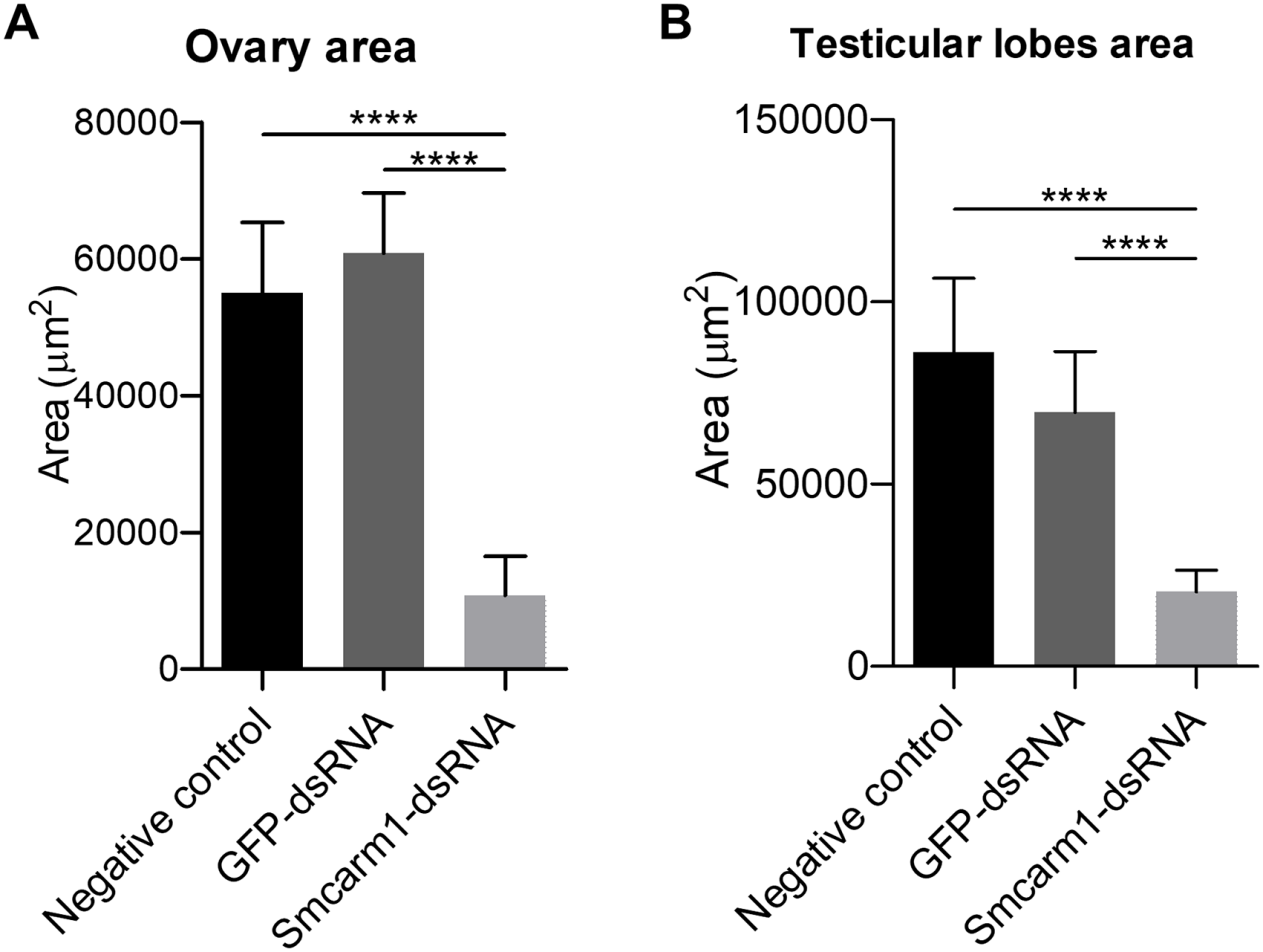
Morphometric analysis of *ex vivo* Sm*carm*1 knocked down adult worms. Morphometric analysis of **(A)** ovary from female and **(B)** testicular lobes from male adult worms recovered from mice infected with schistosomula from negative control (black), unspecific GFP-dsRNA (dark gray), and Sm*carm*1-dsRNA (light gray). Data were generated from three independent experiments and statistically analyzed using the Unpaired *t*-test. ^****^p < 0.0001.

**Figure 6:**
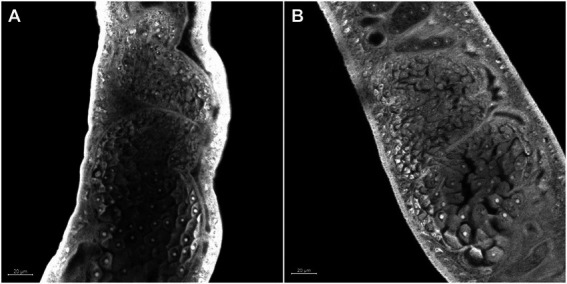
Morphology of the ovary of *ex vivo* Sm*carm*1 knocked-down adult worms. Morphological changes in the ovary of female adult worms recovered from mice infected with schistosomula from **(A)** negative control and **(B)** Sm*carm*1-dsRNA. The white bars indicate the scale 20 μm.

**Figure 7:**
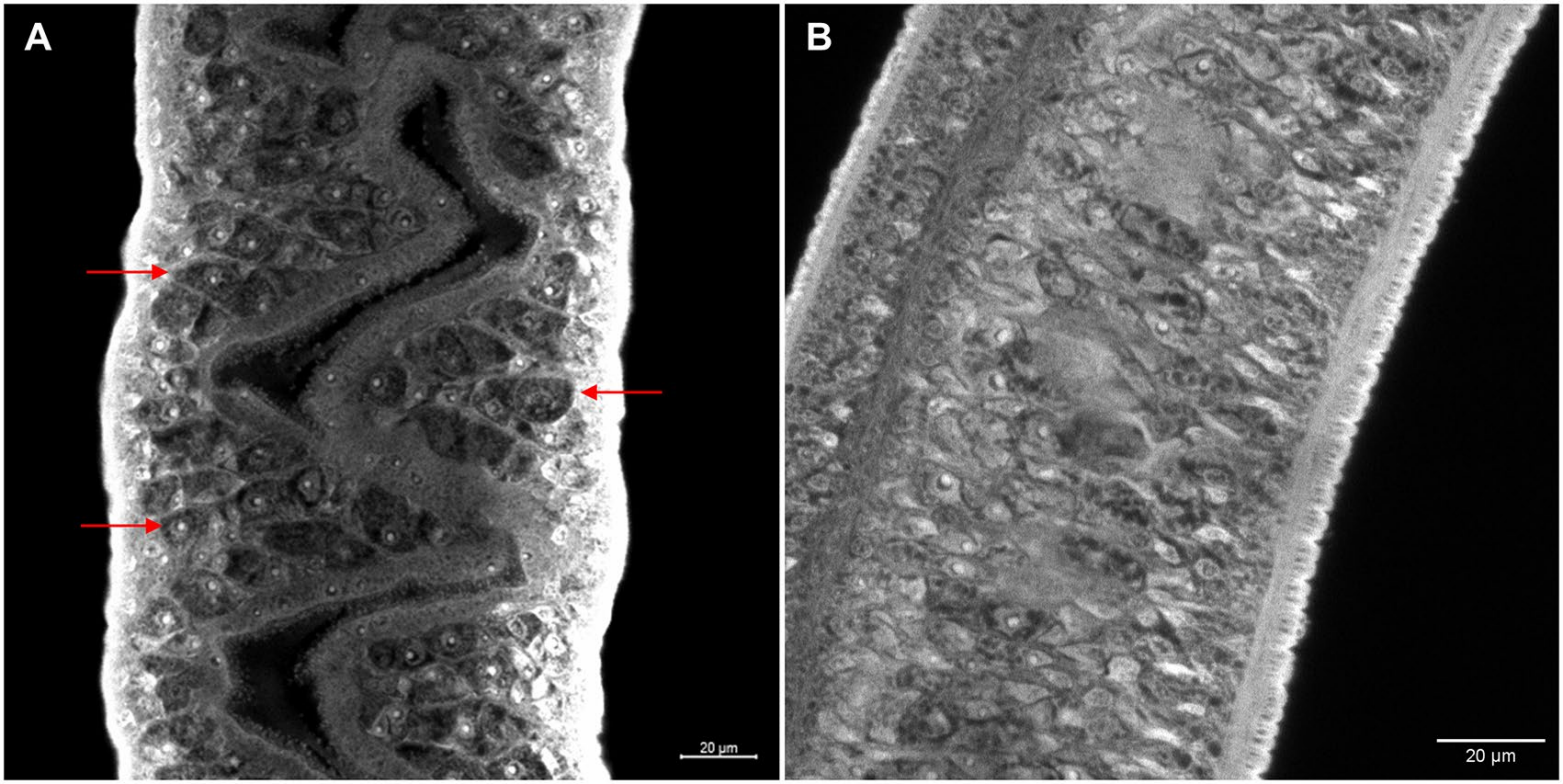
Morphology of *ex vivo* Sm*carm*1 knocked-down female adult worms. Morphological changes in the vitelline glands of female adult worms recovered from mice infected with schistosomula from **(A)** negative control and **(B)** Sm*carm*1-dsRNA. Red arrows highlight the cell organization of the vitelline glands and white bars indicate the scale 20 μm.

Morphometric analyses showed that there was a significant decrease in the ovaries area in females recovered from mice infected with Sm*carm*1 knocked down schistosomula (Figure 5A). In addition to area reduction, it is possible to observe a lower degree of cell differentiation in the ovary (Figure 6) and disorganization of vitelline gland cells (immature cells without the characteristic cluster morphology when compared to the control, which, in turn, presents evident nucleus and characteristic organization of clusters; Figure 7).

Furthermore, there was a decrease in the area of the testicular lobes in male worms recovered from these mice (Figure 5B), however, without a variation in the number of lobes present in each group (data not shown). The testicular lobes were well-demarcated and without the presence of vacuoles (data not shown).

**Figure 8:**
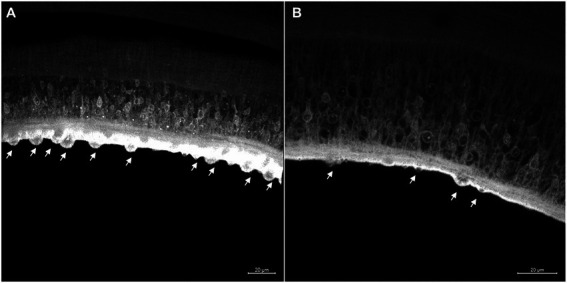
Tegument of *ex vivo* Sm*carm*1 knocked-down male adult worms. Morphological changes in the tegument of male adult worms recovered from mice infected with schistosomula from **(A)** negative control (De Andrade et al., 2014) and **(B)** Sm*carm*1-dsRNA. The arrows indicate the tubercles in the tegument and the white bars indicate the scale 20 μm.

**Figure 9:**
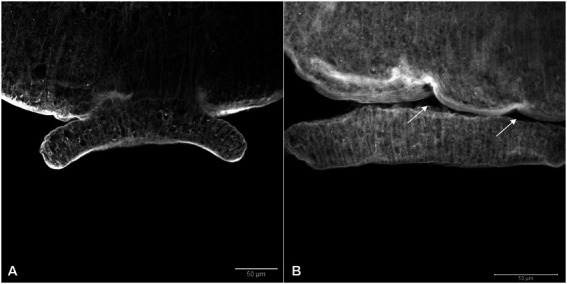
Ventral sucker with irregular tegument in *ex vivo* Sm*carm*1 knocked down adult worms. Morphological changes in the ventral sucker of male adult worms recovered from mice infected with schistosomula from **(A)** negative control and **(B)** Sm*carm*1-dsRNA. The arrows highlight points of the detachment of the ventral sucker to the parasite and white bars indicate the scale 50 μm.

In the tegument of the adult worms recovered from mice infected with Sm*carm*1 knocked-down schistosomula, the presence of fewer tubercles is clearly observed (Figure 8). The ventral sucker of the parasites presented a damaged tegument and a few points of detachment to the parasite (Figure 9).

## Discussion

The protein posttranslational modification (PTM) catalyzed by protein arginine methyltransferases (PRMTs) occurs in cytoplasmic and nuclear proteins. PRMTs can be recruited to gene promoters by transcription factors to methylate arginine residues in the histone tails (Rezai-Zadeh et al., 2003; An et al., 2004). Histone methylation can interfere with the binding of transcriptional effectors and plays diverse roles in regulating chromatin function. In addition, PRMTs can methylate coactivators (Xu et al., 2001; Chevillard-Briet et al., 2002), transcription elongation factors (Kwak et al., 2003; Boulanger et al., 2004), and heterogeneous nuclear ribonucleoproteins (hnRNPs; Xu and Henry, 2004; Yu et al., 2004) also regulating transcriptional initiation and elongation processes and the packaging and export of messenger ribonucleoprotein particles (mRNP; reviewed in Bedford and Richard, 2005). Despite its importance, the methylation of arginine residues catalyzed by PRMTs, whether in histone tails or other proteins, has so far been poorly studied in the schistosome research field.

Publicly available RNAseq data show that the five PRMTs identified in the *S. mansoni* genome are more expressed at the initial stages (18 and 21 days) of infection in male adult worms, despite of the female presence. The expression of Sm*carm*1 in these initial stages could reflect the importance of this protein in the development of male adult worms. Focusing on Sm*carm*1, its expression recovers to similar levels of the early stage (21 dpi) for females from mixed, which is not true for females from single-sex infections. The same pattern of increase in expression is shared by all SmPRMTs, specially Sm*prmt*6, which might indicate a possible role of SmPRMTs in the complete female maturation that could be triggered by the presence of male worms.

Looking at the transcript expression at a single-cell resolution in adult worms (Wendt et al., 2020), we noticed that there is no enrichment in Sm*carm*1 expression at any specific cell clusters, with ubiquitous expressions in almost all cell types, including those related to the reproductive organs, like S1, vitellocytes, and germ cells. In the present work, the reduction of eggs recovered from mice after infection with Sm*carm*1-knockdown schistosomula strengthens the importance of this protein in the regulation of reproduction processes. Indeed, females recovered from mice infected with Sm*carm*1-knocked-down schistosomula presented a significant decrease in the area of the ovaries accompanied by a lower degree of cell differentiation and disorganization of vitelline gland cells. However, Sm*carm*1-knockdown *in vitro* in mature adult worms did not impact egg laying, indicating that Sm*carm*1 is probably more involved in the development of the reproductive organs during worm maturation than in egg formation. The same phenotype was observed with the *in vitro* knockdown of vasa/PL10-like (Sm*vlg1*), a gene related to germline development, in adult females. Mature knocked-down females showed a reduction in the volume of the ovaries, but this did not affect the number of eggs laid (Skinner et al., 2020).

The role of CARM1 in spermiogenesis has already been demonstrated in mice. The *carm*1 knockdown led to low sperm counts and deformed sperm heads showing that the gene is essential for the late stages of germ cell development (Bao et al., 2018). In male worms recovered from mice infected with Sm*carm*1-knockdown schistosomula, we observed a decrease in the area of testicular lobes, which might also influence the observed reduction in egg production.

The tegument of male worms recovered from mice infected with Sm*carm*1-knockdown schistosomula presented fewer tubercles and the ventral sucker presented a damaged tegument and points of detachment from the parasite body. Surprisingly, despite the possibility of a larger exposure of parasite antigens due to tegument damage and the loose ventral sucker adhesion to the worm body, which would impact the parasite adhesion to the host venules, no reduction in parasite numbers was detected. Sm*carm1* expression is enriched in clusters of ambiguous cells in the schistosomula, for which no specific markers or particular processes could be determined, so the role of SmCARM1 in this cell population remains to be elucidated.

Additional studies are necessary to investigate whether the reported effects of Sm*carm*1 knockdown are a consequence of the epigenetic role of SmCARM1 in the methylation of histone tails involved in DNA packaging or other nonhistone proteins associated with transcriptional regulation, splicing mechanism, or mRNA stability.

## Data availability statement

The datasets presented in this study can be found in online repositories. The names of the repository/repositories and accession number(s) can be found in the article/Supplementary material.

## Ethics statement

The animal study was reviewed and approved by Ethics Committee for Animal Use (CEUA) of the Oswaldo Cruz Foundation under license numbers LW13/13 and LW12/16.

## Author contributions

MM, GO, and RP contributed to conception and design of the study. FC, LA, JG, NT, FL, and MM performed the experiments. FC, RN, and JM-S obtained and analyzed the confocal data. SG, FC, and MM performed the statistical analysis. SG performed *in silico* analyses. MM and GO contributed reagents, materials, and analysis tools. FC, SG, and MM wrote the manuscript. All authors contributed to the manuscript revision and approved the submitted version.

## Funding

This work was supported by funding from the European Commission‘s Seventh Framework Program for research, under Grant agreement no. 602080 (A-ParaDDisE), Fundação de Amparo à Pesquisa do Estado de Minas Gerais (FAPEMIG; CBB-APQ-0520-13), and Conselho Nacional de Desenvolvimento Científico e Tecnológico (CNPq; 302518/2018–5 and 317389/2021–1) to MM. Coordenação de Aperfeiçoamento de Pessoal de Nível Superior (CAPES) Programa PCDD-Programa CAPES/Nottingham University (003/2014), CNPq (470673/2014–1, 309312/2012–4, and 304138/2014–2), CAPES (REDE 21/2015), and FAPEMIG (PPM-35 00189–13) to GO. FC and LA fellowships were financed by the CAPES (Finance code 001). SG fellowship was financed by Inova Fiocruz/Fundação Oswaldo Cruz. RP was supported by institutional funds from the CNRS, the Institut Pasteur de Lille, and Lille University.

## Conflict of interest

The authors declare that the research was conducted in the absence of any commercial or financial relationships that could be construed as a potential conflict of interest.

## Publisher’s note

All claims expressed in this article are solely those of the authors and do not necessarily represent those of their affiliated organizations, or those of the publisher, the editors and the reviewers. Any product that may be evaluated in this article, or claim that may be made by its manufacturer, is not guaranteed or endorsed by the publisher.

## Footnotes

^1^ www.schistosomulacellatlas.org

^2^ http://www.genedb.org/Homepage/Smansoni

^3^ http://primer3.sourceforge.net
