# Corrigendum: *Schistosoma mansoni* coactivator associated arginine methyltransferase 1 (SmCARM1) effect on parasite reproduction

**DOI:** 10.3389/fmicb.2023.1190341

**Published:** 2023-03-29

**Authors:** Fernanda Sales Coelho, Sandra Grossi Gava, Luiza Freire Andrade, Juliana Assis Geraldo, Naiara Clemente Tavares, Felipe Miguel Nery Lunkes, Renata Heisler Neves, José Roberto Machado-Silva, Raymond J. Pierce, Guilherme Oliveira, Marina Moraes Mourão

**Affiliations:** ^1^Grupo de Pesquisa em Helmintologia e Malacologia Médica, Instituto René Rachou, Fundação Oswaldo Cruz—FIOCRUZ, Belo Horizonte, Minas Gerais, Brazil; ^2^Department of Clinical Research, London School of Hygiene and Tropical Medicine, London, United Kingdom; ^3^Laboratório de Helmintologia Romero Lascasas Porto, Departamento de Microbiologia, Imunologia e Parasitologia, Faculdade de Ciências Médicas, Universidade do Estado do Rio de Janeiro, Rio de Janeiro, Brazil; ^4^Université de Lille, CNRS, Inserm, CHU Lille, Institut Pasteur de Lille, U1019—UMR 9017—CIIL—Centre d'Infection et d'Immunité de Lille, Lille, France; ^5^Instituto Tecnológico Vale, Belém, Pará, Brazil

**Keywords:** *Schistosoma mansoni*, coactivator associated arginine methyltransferase 1, SmCARM1, epigenetics, reproduction

In the published article, there was an error. In the section Materials and methods, “*In silico* analyses,” paragraph 1, it was previously stated that:

“Additionally, we checked the SmPRMTs expression in a publicly available RNAseq dataset (Protasio et al., 2017) retrieved from WormBase ParaSite as the median of transcripts per million units (TPMs) per gene.”

The corrected sentence appears below:

“Additionally, we checked the SmPRMTs expression in a publicly available RNAseq dataset (Protasio et al., 2017) retrieved from WormBase ParaSite as counts of aligned reads per run per gene.”

The authors apologize for this error and state that this does not change the scientific conclusions of the article in any way. The original article has been updated.
