# Supplementary material for: Schistosoma mansoni coactivator associated arginine methyltransferase 1 (SmCARM1) effect on parasite reproduction
Source: Front Microbiol. 2023 Feb 24;14:1079855. doi: 10.3389/fmicb.2023.1079855 (PMC9998485; doi:10.3389/fmicb.2023.1079855)
Supplement: Supplementary file 1 [file Data_Sheet_1.PDF]

Supplementary Material

Supplementary Tables S1. Expression patterns of PRMTs in *Schistosoma mansoni*. Expression patterns of PRMTs in females (F) and males (M), of single (SS) and mixed (MS) infections. Columns indicate effect size estimate (log2FoldChange) and Benjamini-Hochberg (BH) adjusted p-values (*padj*), for 18-, 28-, 35-, and 38-days post-infection (dpi) relative to 21 dpi. Statically significant values (*padj* < 0.05) are highlighted in bold. EXCEL FILE.

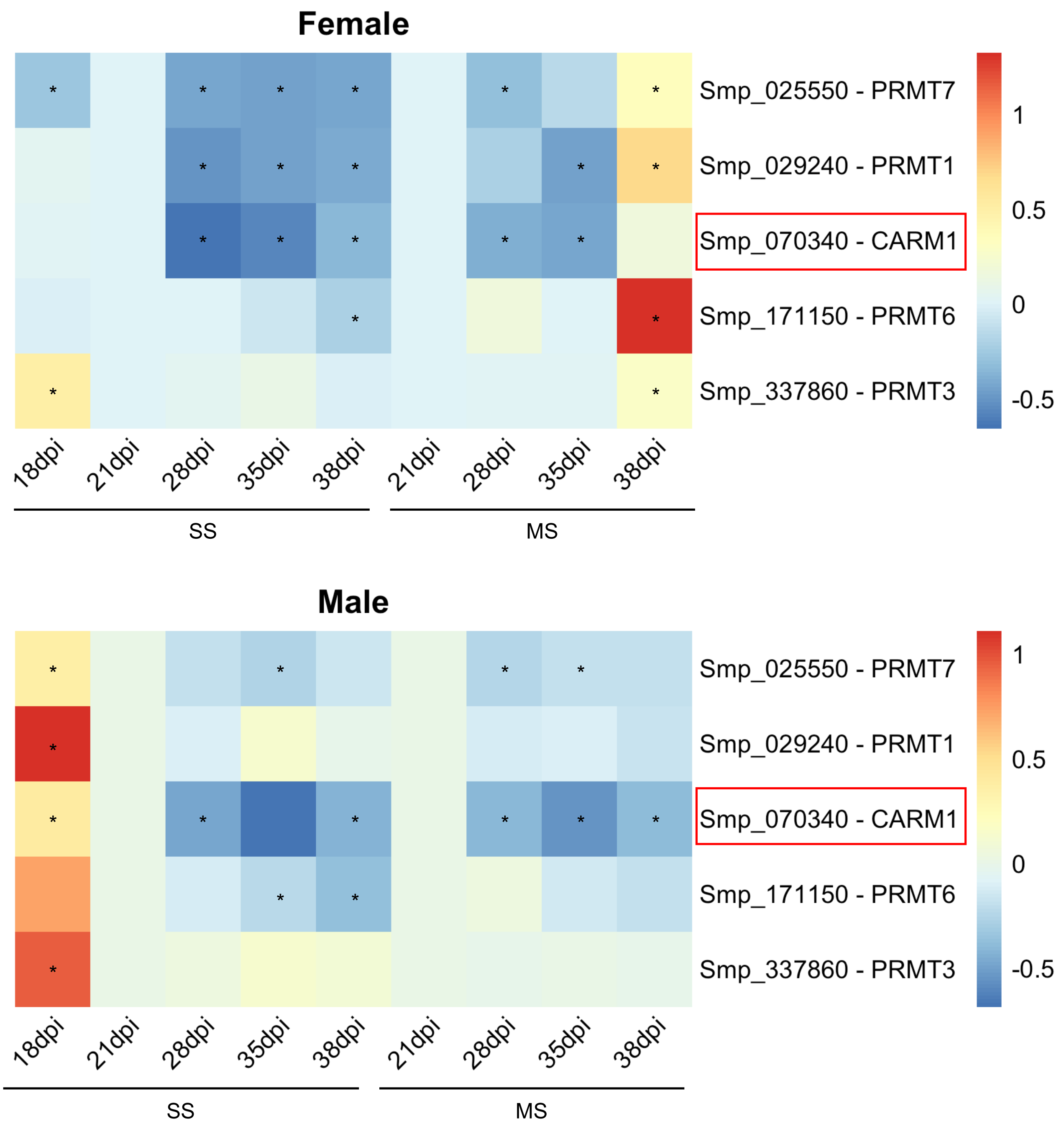

Supplementary Figure S1. Expression patterns of SmPRMTs in *Schistosoma mansoni*. Expression patterns of SmPRMTs in females and males, of single (SS) and mixed (MS) infections, after 18-, 21-, 28-, 35-, and 38-days of infection (dpi). The columns indicate the life stages of the parasite, and the lines display the different PRMTs. The color scale indicates higher (red) or lower (blue) expression of the analyzed transcripts based on log2 Fold Change values for 18-, 28-, 35-, and 38-days post-infection (dpi) relative to 21 dpi. Sm*carm*1 (Smp_070340) is highlighted by a red box. Statically significant changes (*padj* < 0.05) are represented with asterisks.

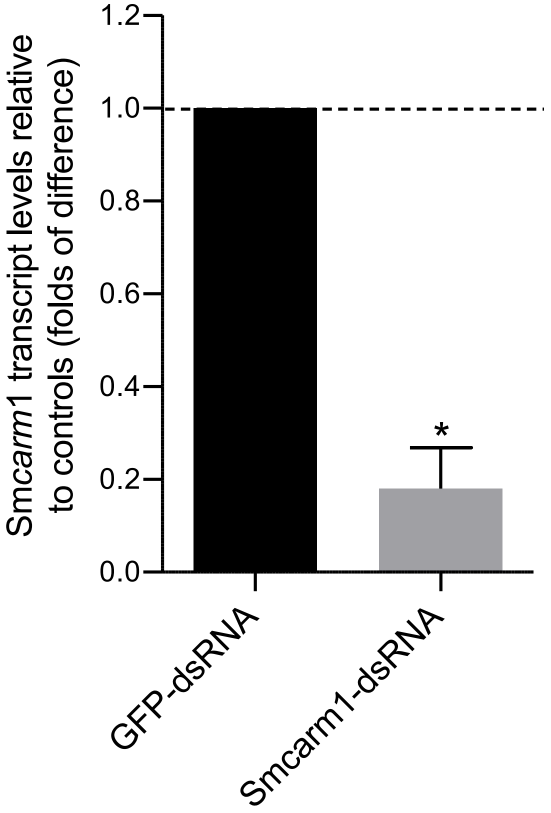

Supplementary Figure S2. Sm*carm*1 transcript levels in schistosomula before mice infection. Bar graph depicting the Sm*carm*1 transcript levels in schistosomula after two days of exposure to unspecific GFP-dsRNA (black) or Sm*carm*1-dsRNA (gray) relative to controls. Data are represented as mean fold-difference (±SE) relative to controls (dashed line). Mann-Whitney test. *$p<0.05$.
